# Posttraumatic venous gas in the liver – a case report and review of the current literature

**DOI:** 10.1186/s12893-018-0345-z

**Published:** 2018-03-02

**Authors:** René Fahrner, Falk Rauchfuss, Hubert Scheuerlein, Utz Settmacher

**Affiliations:** 1 University Hospital Jena, Division of General, Visceral and Vascular Surgery, Am Klinikum 1, 07740 Jena, Germany; 2 St. Vincenz Hospital, Division of General and Visceral Surgery, Am Busdorf 2, 33098 Paderborn, Germany

**Keywords:** Abdominal trauma, Gas, Liver vein

## Abstract

**Background:**

There are numerous causes of hepatic gas formation that range from serious pathologies to incidental findings, including mesenteric infarction, liver abscess, inflammatory bowel disease or minimally invasive hepatic interventions.

**Case presentation:**

We report a case of a 50-year-old man who was admitted to the emergency room after a car accident. The clinical examination and further diagnostics revealed a craniocerebral injury with a fracture of the skull, concomitant soft tissue lesions and subarachnoidal bleeding. Furthermore, a blunt thoracic trauma with hemopneumothorax due to rib fractures was treated with a chest tube. No obvious abdominal pathology was seen. While in the operating theatre for the surgical revision of the cranial soft tissue lesions, a femoral venous catheter was inserted without any complications. A routine ultrasound of the abdomen six hours after the trauma revealed unclear hepatic gas formation. A contrast-enhanced computer tomography (CT) scan of the abdomen was performed, and the gas formation was found to be localized within the left hepatic vein. Afterwards, there was no specific treatment of the hepatic venous gas formation, as no alterations of liver function or liver enzymes were seen. The further course of the patient was uneventful regarding the gas formation in the liver, and another ultrasound two days later revealed no further gas in the liver.

**Conclusions:**

The placement of a femoral venous catheter is a risk factor for gas formation in liver veins. No further treatment is needed in cases with stable liver function. To rule out serious pathologies, diagnostic findings (e.g., ultrasound, CT), clinical history and underlying diseases need to be analyzed carefully after the detection of intrahepatic gas formation. With contrast-enhanced CT, the localization of the gas and its potential causes might be detectable.

## Background

There are numerous causes of hepatic gas formation [1]. They range from serious pathologies, such as mesenteric infarction, liver abscess, inflammatory bowel disease, thrombosis of a hepatic artery after liver transplantation, cholecystitis and cholangitis, to incidental findings without any consequences. Furthermore, minimally invasive hepatic interventions (endoscopic retrograde cholangiopancreatography, hepatic artery embolization, tumour ablation, liver biopsy, portal vein embolization) are increasing in frequency, so it is important to rule out these procedures as potential causes of the gas formation in order to detect life-threatening conditions in the patient. Therefore, the patient’s history and the radiological findings need to be analyzed carefully. We report here a case of hepatic gas formation after a motor vehicle crash with suspected blunt abdominal trauma and discuss the potential diagnosis.

## Case presentation

**Fig. 1:**
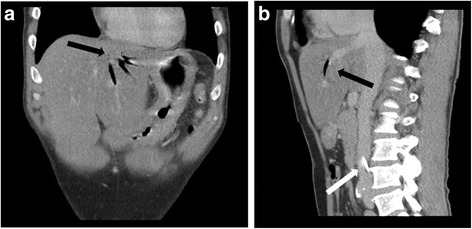
Contrast-enhanced CT scan of the abdomen several hours after a car crash with suspected blunt abdominal trauma showing gas formation within the left liver vein (black arrow **a**, **b**). Furthermore, the central venous catheter is seen in the vena cava inferior (white arrow, **b**)

A 50-year-old man was admitted to the emergency room after a car crash. At the time of admission, the patient was conscious and hemodynamically stable. Therefore, a computer tomography (CT) scan was performed for further diagnostics and revealed a craniocerebral injury that involved a fracture of the skull with concomitant soft tissue lesions and subarachnoidal bleeding. In addition, the patient suffered blunt thoracic trauma with hemopneumothorax due to rib fractures, which was treated with a chest tube. No obvious abdominal pathology was seen. The patient was transferred to the operating theatre for surgical revision of the soft tissue lesions. During general anesthesia, a femoral venous catheter was inserted without any procedural complications. Postoperatively, the patient was monitored in the intensive care unit. At our institution, a repeated ultrasound of the abdomen is performed six hours after the trauma to rule out free abdominal liquid or lesions of the parenchymatous organs, because it has been shown that repeated ultrasound examinations decrease the rate of false negative results and increase the sensitivity to detect intraabdominal liquid [2, 3]. In our patient, an unclear hepatic gas formation in the liver veins was seen but still no free liquid. For further differentiation and localization of the gas formation, a contrast-enhanced CT scan of the abdomen was performed. Here, the gas formation was localized in the left hepatic vein (Fig. 1 a/b, black arrow). In addition, the central venous catheter in the inferior vena cava was visible without hematoma or gas formation (Fig. 1 b, white arrow), and no additional abdominal pathology was noted.

There was no specific treatment administered for the hepatic venous gas formation. The further course of the patient was uneventful regarding the gas formation in the liver, and an additional ultrasound to check for further gas formation two days later revealed no more gas in the liver. No changes in liver function or liver enzymes were seen.

## Discussion und conclusions

Gas formation within the hepatic veins is rarely seen in routine CT scans, and only a few cases have been reported so far. The largest published investigation of patients with gas formation in the liver vein analyzed 235 CT scans of intensive care unit patients and found that 5% of the CT scans showed gas formation in the hepatic veins [4]. All patients with gas in their hepatic veins in this study had received a femoral venous catheter. Therefore, the authors concluded that a femoral venous catheter is one of the most important risk factors for this complication. Further, they recommended performing a multiplanar reconstruction of the liver vascular structures to identify the localization of the gas formation. Portal venous gas [5, 6], pneumobilia [7] or gas in the liver artery [8] were considered as differential diagnoses and lead to further diagnostics and therapy. In postmortem studies, cardiac gas embolism, putrefaction processes, and open skull fractures due to craniocerebral injury or gunshot injury to the head were associated with gas in the hepatic veins [9–11]. In addition, gas embolism in the hepatic vein during laparoscopic liver surgery has been reported [12–14]. As interventional therapies of the liver, such as endoscopic retrograde cholangiopancreatography, hepatic artery embolization, tumour ablation, liver biopsy, and portal vein embolization, are increasing in number, post-interventional complications with gas within the liver are seen more frequently.

In this report, the further course of the patient was uneventful, and a subsequent ultrasound revealed no more gas in the liver, and no changes in liver function were detected. Most likely, the detection of hepatic venous gas formation in our patient was a coincidence related to the placement of the femoral venous catheter and the open skull fracture. This might explain why the initial CT scan was unremarkable and showed no pathology of the liver.

It has been shown that focused abdominal sonography after blunt abdominal trauma is a reliable and sensitive technique in the hands of experienced investigators [3, 15]. In the case of negative findings, repeated examinations may help decrease the rate of false negative results and increase the sensitivity to detect free abdominal liquid [2, 3, 15]. Intravascular abdominal gas after blunt abdominal trauma is rarely seen and is postulated to be a transient finding that can often be treated non-surgically [16, 17]. In total, posttraumatic hepatic gas formations have been reported more frequently within the portal venous system than within the hepatic venous system.

Portal venous gas in combination with pneumatosis intestinalis might be a sign of intestinal ischemia but might also occur after blunt abdominal trauma or abdominal surgery [5, 6, 17, 18]. In this patient, there was no evidence of intestinal ischemia from the clinical examination or the CT scan. Pneumobilia is reported as a consequence of blunt abdominal trauma [7], but in our patient, this pathology was not supported by the localization of the gas formation. Infectious and abscess-forming complications after liver transplantation [8] or liver abscess [19] with gas in the hepatic artery are rare, and they were not considered reasonable diagnoses in our patient due to the short-time follow-up. As the patients’ history was uneventful until the car crash, complications of minimally invasive interventions to the liver were also not reasonable.

The causes of hepatic gas formation might range from serious pathologies to incidental, harmless findings. Therefore, it is important to analyze and clarify the underlying cause of hepatic gas formation when it is identified. Multiplanar reconstructions of the vascular structures of the liver by CT scan have been recommended as a means of identifying the exact localization of the gas formation within the liver. Depending on the diagnosis, further treatment may be necessary. Gas formation within the hepatic veins is rare and usually harmless, as in our case, without the need for medical or surgical treatment.

## Abbreviations

CT: Computer tomography
